# Physiological and Transcriptomic Responses of *Arthrospira platensis* to Low-Density Polyethylene Microplastic Exposure

**DOI:** 10.3390/biology15080653

**Published:** 2026-04-20

**Authors:** Sekbunkorn Treenarat, Authen Promariya, Wuttinun Raksajit

**Affiliations:** 1 Program of Animal Health Technology, Faculty of Veterinary Technology, Kasetsart University, Bangkok 10900, Thailand; sekbunkorn.tr@ku.th (S.T.); authen.pr@ku.th (A.P.); 2 Department of Veterinary Nursing, Faculty of Veterinary Technology, Kasetsart University, Bangkok 10900, Thailand

**Keywords:** *Arthrospira platensis*, gene ontology, LDPE microplastics, photosynthetic pigments, transcriptomic analysis

## Abstract

Microplastics (MPs), particularly low-density polyethylene (LDPE), are widespread pollutants in aquatic environments and may affect cyanobacterial physiology. This study investigated the concentration-dependent effects of LDPE-MPs on the physiology and transcriptomic responses of *Arthrospira platensis*. Cultures were exposed to 10–5000 mg/L LDPE-MPs (nominal size ≤ 500 µm) for 16 days. Low to moderate concentrations (10–1000 mg/L) produced minimal effects on growth, biomass accumulation, or pigment contents. In contrast, higher concentrations (3000–5000 mg/L) were associated with reduced growth and biomass, accompanied by declines in chlorophyll *a* (Chl *a*) and phycobiliproteins over time. By day 16 at 5000 mg/L, biomass and Chl *a* decreased to 1.47 ± 0.03 g/L and 8.39 ± 0.24 µg/mL, respectively, compared with 1.64 ± 0.04 g/L and 10.81 ± 0.52 µg/mL in the control (*p* < 0.05). Accordingly, Chl *a* yield decreased by 13%. Field-emission scanning electron microscopy revealed adhesion of LDPE particles to filament surfaces and the formation of extracellular polymeric substance (EPS)-rich aggregates, which may influence light availability and nutrient exchange. Transcriptomic analysis indicated changes in several metabolic pathways, including nitrogen assimilation, photosynthetic electron transport, carbon metabolism, and metal homeostasis, together with differential expression of genes related to stress responses and EPS biosynthesis. Overall, these findings suggest that relatively high concentrations of LDPE microplastics may influence physiological and metabolic processes in *A. platensis*.

## Simple Summary

Microplastics are increasingly found in aquatic environments, but their effects on cyanobacteria are not yet well understood. This study examined how the cyanobacterium *Arthrospira platensis* responds to short-term exposure to low-density polyethylene (LDPE) microplastics at different concentrations. Changes in growth, biomass, and photosynthetic pigments (chlorophyll *a*, phycocyanin, and allophycocyanin) were measured during the exposure period. Low to moderate concentrations produced minimal effects, whereas higher concentrations were associated with reduced growth, biomass, and pigment contents over time. Cell morphology and interactions between microplastics and the cell surface were also observed using electron microscopy. In addition, RNA sequencing was used to explore changes in gene expression related to stress responses, pigment synthesis, and cellular metabolism. The results provide insight into how *A. platensis* responds to microplastic exposure under controlled experimental conditions.

## 1. Introduction

The rapid increase in plastic production has led to widespread plastic accumulation in aquatic environments, raising serious concerns about its environmental impacts. Plastic debris progressively fragments into particles smaller than 5 mm, known as microplastics (MPs) [1]. Although oceans were long considered the primary global sink for plastic waste, recent studies have indicated that terrestrial ecosystems, particularly soils, also represent major reservoirs of MPs [2]. In aquatic systems, the discharge of untreated wastewater containing MPs can intensify organic pollution, reduce dissolved oxygen, and promote eutrophication, thereby degrading water quality [3]. Despite these concerns, wastewater treatment efforts remain insufficient, as MPs frequently bypass filtration processes, resulting in their continuous release into the environment [4]. As a result, microplastics derived from commonly used polymers, such as polyethylene (PE), polypropylene (PP), and polyethylene terephthalate (PET), are now ubiquitous and have been detected across marine, freshwater, and polar ecosystems worldwide, including oceans, rivers, and lakes [5,6]. Among these polymers, low-density polyethylene (LDPE) is a thermoplastic polymer derived from the monomer ethylene and is recognized for its flexibility, chemical resistance, and durability [7]. Because of its low density, affordability, and ease of processing, LDPE is widely used in everyday products such as plastic bags, food packaging, bottles, containers, and cable coatings, making it an essential material across industries including consumer goods, agriculture, and healthcare [8]. However, LDPE presents serious environmental challenges because it is non-biodegradable. Once released into the environment, LDPE products can persist for hundreds of years. Over time, exposure to sunlight, mechanical stress, and other environmental factors cause LDPE to fragment into MPs. Consequently, the extensive use and improper disposal of LDPE lead to its accumulation in soils, waterways, and even the atmosphere, posing persistent challenges for waste management and environmental conservation [9]. MPs are primarily derived from land-based activities and are subsequently transported into river systems, leading to relatively higher loads in freshwater compared with marine environments [10]. This pattern may also reflect the lower water volumes in freshwater systems, which limit dilution. Environmental MP concentrations are commonly reported as particle counts [11], typically ranging from 1 to 846 particles/L in freshwater [12] and 0.002–16,272 particles/m^3^ in marine environments [13], or as mass concentrations of approximately 0.31–900 mg/L in freshwater, depending on location and particle size fraction [14,15]. In addition, actual MP abundance in aquatic environments is likely underestimated, given the ongoing limitations of analytical techniques for precise detection and quantification in complex environmental matrices.

Microalgae and cyanobacteria are among the organisms affected by microplastic accumulation in aquatic environments [16]. These photosynthetic microorganisms convert CO_2_ into energy and release oxygen, while also contributing to nitrogen removal in wastewater through their rapid growth and high biomass production. In addition, both microalgae and cyanobacteria produce a wide range of secondary metabolites, including pigments, proteins, carbohydrates, and antioxidants, which are widely utilized in agriculture as animal feed and in the pharmaceutical industry as medicinal and nutritional supplements [17,18]. Despite their ecological and biotechnological importance, microalgae and cyanobacteria are frequently exposed to emerging stressors such as MPs, yet their effects remain inconsistently reported, with the same polymer shown to stimulate, inhibit, or have no effect on growth depending on species and experimental conditions [19].

*Arthrospira platensis* is an economically important cyanobacterium widely used as a food supplement and feed additive due to its high nutritional value and diverse bioactive properties [17,18]. The increasing prevalence of MPs in aquatic environments poses a potential risk to its large-scale cultivation, particularly in open systems susceptible to plastic-derived contamination [10]. Among MPs, LDPE is one of the most abundant polymers in aquatic ecosystems; however, its effects on the growth and physiological performance of *A. platensis* remain poorly understood. Previous studies on *Arthrospira* sp. and other microalgae have generally examined microplastic concentrations up to 900 mg/L [15,20], which already exceed typical environmental levels. Accordingly, higher concentrations (1000–5000 mg/L) are used in laboratory experiments to evaluate potential toxic responses under extreme exposure conditions rather than to represent natural environments. To our knowledge, no previous study has comprehensively investigated gene expression responses in *Arthrospira* sp. at microplastic concentrations exceeding 1000 mg/L. Thus, this work provides new molecular insights into cyanobacterial stress responses under elevated microplastic exposure. Accordingly, we investigated the concentration- and time-dependent effects of LDPE microplastics (LDPE-MPs) on growth, dry biomass, and pigment composition. Controlled cultivation experiments, combined with light microscopy, field-emission scanning electron microscopy (FE-SEM), and RNA sequencing, were performed to evaluate cell surface alterations and elucidate the underlying molecular mechanisms.

## 2. Materials and Methods

### 2.1. Cultivation and Microplastic Exposure of A. platensis

*A. platensis* strain IFRPD1182 was obtained from the Institute of Food Research and Product Development, Kasetsart University, Bangkok, Thailand. It was cultured in 250 mL Erlenmeyer flasks containing 100 mL of Zarrouk medium (pH 10.0), with an initial optical density of 0.1 at 730 nm (OD_730_), as measured using a spectrophotometer. The medium composition was as follows: 16.8 g/L of NaHCO_3_, 2.5 g/L of NaNO_3_, 1.0 g/L of K_2_SO_4_, 1.0 g/L of NaCl, 0.5 g/L of K_2_HPO_4_, 0.04 g/L of CaCl_2_·2H_2_O, 0.03 g/L of Na_2_EDTA·2H_2_O, 0.2 g/L of MgSO_4_·7H_2_O, and 0.01 g/L FeSO_4_·7H_2_O. It was supplemented with 1.0 mL/L of a trace element solution with the following composition: 2.86 g/L of H_3_BO_3_, 0.02 g/L of MoO_3_, 1.81 g/L of MnCl_2_·4H_2_O, 0.08 g/L of CuSO_4_·5H_2_O, 0.22 g/L of ZnSO_4_·7H_2_O, and 0.05 g/L of Co(NO_3_)_2_·6H_2_O. Cultures were incubated under continuous illumination (40 μmol photons/m^2^/s) at 32 °C ± 2 °C with agitation at 120 rpm. Cell cultures were prepared in triplicate under six LDPE-MP exposure conditions: a control (no LDPE-MPs) and five LDPE-MP concentrations (10, 100, 1000, 3000, and 5000 mg/L). LDPE-MP powder (nominal maximum size ≤ 500 μm, exhibiting a heterogeneous size distribution; CAS-No:9002-88-4) were purchased from Thermo scientific (Thermo Fisher Scientific, Waltham, MA, USA; catalog no. A10239.22) and used in this study. Before the experiment, the LDPE-MP powder was surface-sterilized by exposure to UV radiation ($\lambda_{max}$ = 254 nm, 20 cm, 0.5 mW/cm^2^) [21]. After sterilization, residual bacterial contamination on LDPE-MPs was assessed using standard microbiological procedures [22].

### 2.2. Growth Monitoring, Biomass Determination, and Pigment Analysis

Growth was monitored during the cultivation period by measuring the OD_730_ value daily. The recorded OD_730_ values served as indicators of the growth performance of *A. platensis* throughout the experiment [23]. After 16 days, the culture reached the logarithmic growth phase, with an OD_730_ value of 0.8–1.0. The cells were harvested by filtration using 0.45 µm cellulose nitrate membranes and subsequently rinsed with distilled water to eliminate remaining salts. The collected biomass was then dried in an oven at 80 °C for 24 h, and biomass yield was reported as grams per liter (g/L) [24].

Chlorophyll *a* (Chl *a*) content was determined according to Equation (1). Briefly, cells were transferred to microtubes containing absolute methanol and incubated in the dark at 4 °C for 20 min. Next, the extracts were centrifuged at 16,000× *g* for 15 min. The absorbance of the supernatant was then measured spectrophotometrically at 665 nm (λ_max_ for Chl *a*_,_ A_665_) and 720 nm (background turbidity, A_720_) using a Genesys 30 spectrophotometer (Thermo Fisher Scientific, Waltham, MA, USA) to quantify Chl *a* content, as described by [25,26]. The equations are as follows:

(1) $$Chl\;a(\mu g/mL)=12.9447\times (A_{665}-A_{720})$$

Phycocyanin (PC) and allophycocyanin (APC) contents were determined according to Equations (2) and (3). Briefly, cells were suspended in phosphate buffer (pH 7.0) containing 1.5% (*w*/*v*) CaCl_2_ and disrupted using an ultrasonic homogenizer (Thermo Fisher Scientific, Waltham, MA, USA). After incubation, the homogenate was centrifuged at 16,000× *g* for 15 min. The absorbance of the resulting supernatant was then measured spectrophotometrically at 620 nm (λ_max_ for PC, A_620_) and 652 nm (λ_max_ for APC, A_652_) using a Genesys 30 spectrophotometer (Thermo Fisher Scientific, Waltham, MA, USA), as described by [27,28]. The equations are as follows:

(2) $$PC(\mu g/mL)=\frac{\left[(A_{620}-[0.474\times A_{652}]\right)}{5.34}$$

(3) $$APC(\mu g/mL)=\frac{\left[(A_{652}-[0.208\times A_{620}]\right)}{5.09}$$

The yield of each pigment (Chl *a*, PC, and APC), expressed as mg/g, was calculated according to Equation (4) by dividing the measured pigment concentration by the dry biomass concentration of *A. platensis*. The equation is as follows:

(4) $$Pigment\;yield(mg/g)=\frac{Pigment\;concentration\;(\mu g/mL)}{Biomass\;concentration(g/L)}$$

### 2.3. Light Microscopy and FE-SEM Analysis

The morphological characteristics of *A. platensis* were examined using a Carl Zeiss binocular light microscope at 100× magnification and field-emission scanning electron microscopy (FE-SEM). For FE-SEM, cells were fixed in 2.5% glutaraldehyde at 4 °C for 24 h and then washed with distilled water. Next, a 50 μL aliquot of the suspension was placed on 12 mm round glass coverslips and allowed to adhere at room temperature for 15–20 s. Then, the samples were dehydrated through a graded ethanol series (30%, 50%, 70%, 80%, and 90% for 15 min each, followed by 100% ethanol twice for 20 min each), mounted in a single layer on conductive adhesive stubs, sputter-coated with gold, and critical-point dried. FE-SEM images were then captured at an accelerating voltage of 15 kV with a working distance of 1–5 μm.

### 2.4. Total RNA Extraction, Library Preparation, and RNA-Sequencing

Among the tested treatments, the 5000 mg/L condition was selected for transcriptomic analysis. Cultures of *A. platensis* were sampled on day 6, and three independent biological replicates were prepared for each treatment. Total RNA was extracted from independent cultures using the Quick-RNA Miniprep Plus Kit (Zymo Research, Irvine, CA, USA) prior to sequencing. Briefly, cells were harvested by centrifugation at 16,000× *g* and 25 °C for 1 min, resuspended in 1 × DNA/RNA Shield, and homogenized by ultrasonication on ice. Next, total RNA was purified through filtration, lysis, ethanol precipitation, column binding, and DNase I treatment. After washing, total RNA was eluted with DNase/RNase-free water. RNA concentration and integrity were assessed using the Agilent 2100 Bioanalyzer and 1% agarose gel electrophoresis, where clear 23S and 16S rRNA bands and Bioanalyzer electropherogram profiles confirmed good RNA integrity based on the RNA Integrity Number (RIN) algorithm [29] and suitability for library preparation [29]. For library preparation, 300–500 ng of total RNA was subjected to rRNA depletion using DNA probes, followed by enzymatic digestion. The remaining RNA was fragmented with divalent cations and reverse-transcribed into cDNA using random primers. dUTP was incorporated during second-strand synthesis, followed by adapter ligation, purification, and PCR amplification using P5/P7 primers and uracil-DNA glycosylase. Indexed libraries were validated and sequenced using 150-bp paired-end reads on the Illumina NovaSeq 6000 platform. Raw sequencing reads were quality-trimmed using Trimmomatic-0.33 [30].

### 2.5. Differential Gene Expression Analysis

Differential gene expression analysis was performed using DESeq2 (version 1.26.0) with a screening threshold of *p* < 0.05 and |log_2_FoldChange| > 1 [31]. The identified differentially expressed genes were further analyzed using a volcano plot and subjected to functional enrichment analyses. Enrichment analyses, including Gene Ontology (GO) and KEGG Pathway enrichment, were conducted on the differentially expressed gene sets using GOseq (version 1.10.0) [32], and the ClusterProfiler R package (version 3.14.3) [33].

### 2.6. Functional Annotation of Genes

Functional annotation of genes was performed by comparing the clusters of orthologous groups (COGs) identified in the genomes with those from reference genomes available in the National Center for Biotechnology Information (NCBI) database (https://www.ncbi.nlm.nih.gov/research/cog/, accessed on 31 January 2025) to ensure a comprehensive functional assessment.

### 2.7. Statistical Analysis

All data presented in this study represent the means of three independent biological replicates, with error bars indicating the standard deviation (Mean ± SD, *n* = 3). Statistical analyses were performed using one-way analysis of variance (ANOVA), and significant differences (*p* < 0.05) were determined using Tukey’s HSD test in SPSS version 22 (IBM Corp., Armonk, NY, USA).

## 3. Results

### 3.1. Effect of LDPE-MPs on the Growth and Biomass of A. platensis

**Figure 1:**
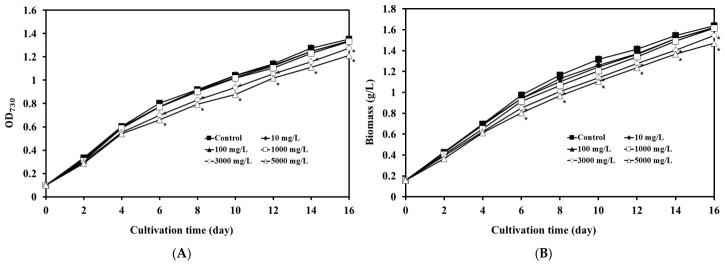
Effects of different LDPE-MPs concentrations on the growth and biomass of *A. platensis* over time (**A**) OD_730_ values and (**B**) Biomass. Each point in a line graph represents mean values (±SD, *n* = 3) per treatment. An asterisk (*) indicates a statistically significant difference from the control on each cultivation day (*p* < 0.05).

*A. platensis* was cultured in the presence of varying concentrations of LDPE-MPs (10, 100, 1000, 3000, and 5000 mg/L) (Figure 1A). The growth curves (OD_730_) showed no significant differences throughout the cultivation period at LDPE-MPs concentrations ranging from 0 to 1000 mg/L. In contrast, higher LDPE-MP concentrations (3000 and 5000 mg/L) resulted in significantly reduced growth compared with the control (*p* < 0.05), with the reduction becoming evident from day 6 and average OD_730_ values of 1.27 and 1.21, respectively, on day 16. In addition, the initial average dry biomass across all tested conditions was 0.16 ± 0.03 g/L (Figure 1B). On day 16, the average dry biomass did not differ significantly between the control (1.64 ± 0.04 g/L) and the 10–1000 mg/L treatments, but was significantly lower in the 3000 and 5000 mg/L treatment groups (1.54 ± 0.04 and 1.47 ± 0.04 g/L, respectively) (*p* < 0.05), with a clear decline in biomass observed from day 6 onward.

### 3.2. Effect of LDPE-MPs on the Pigment Production of A. platensis

**Figure 2:**
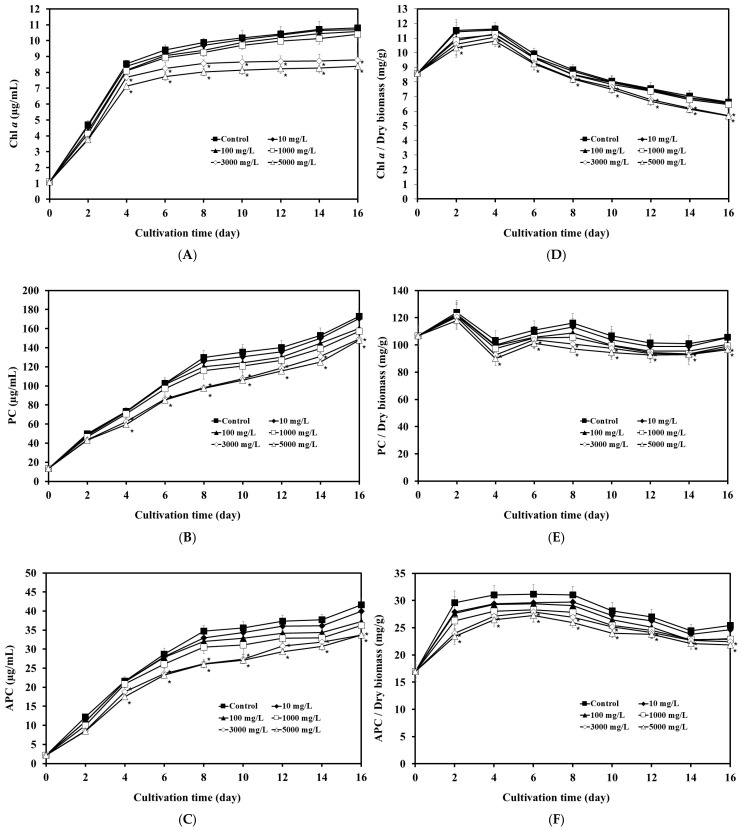
Effects of different LDPE-MP concentrations on pigment contents and pigment yields of *A. platensis* over time. Panels (**A**–**C**) show pigment contents (µg/mL): (**A**) chlorophyll *a* (Chl *a*), (**B**) phycocyanin (PC), and (**C**) allophycocyanin (APC). Panels (**D**–**F**) show pigment yields (mg/g dry biomass): (**D**) Chl *a*, (**E**) PC, and (**F**) APC. Each point represents the mean ± SD (*n* = 3). An asterisk (*) indicates a statistically significant difference from the control on the corresponding cultivation day (*p* < 0.05).

Exposure to LDPE-MPs affected the synthesis of photosynthetic pigments, including chlorophyll *a* (Chl *a*) (Figure 2A). Chl *a* content remained comparable to the control at 10–1000 mg/L, but declined significantly at higher concentrations (3000 and 5000 mg/L) from day 4 onward. By day 16, Chl *a* concentration decreased to 8.82 ± 0.43 and 8.39 ± 0.24 µg/mL at 3000 and 5000 mg/L, respectively, compared with 10.81 ± 0.52 µg/mL in the control (*p* < 0.05). Consistent with this trend (Figure 2D), Chl *a* yield declined after day 4, reaching 5.73 ± 0.31 and 5.71 ± 0.21 mg/g at 3000 and 5000 mg/L, respectively, compared with 6.58 ± 0.36 mg/g in the control (*p* < 0.05). Similarly, phycobiliproteins, including phycocyanin (PC) and allophycocyanin (APC), were affected by LDPE-MP exposure (Figure 2B,C). Their concentrations remained comparable to the control at up to 1000 mg/L but declined significantly at higher concentrations (3000 and 5000 mg/L) from day 4 onward. By day 16, PC concentrations decreased to 150.04 ± 1.31 and 141.71 ± 1.77 µg/mL at 3000 and 5000 mg/L, respectively, compared with 173.27 ± 1.68 µg/mL in the control (*p* < 0.05). Similarly, APC concentrations declined to 34.57 ± 1.48 and 32.01 ± 1.46 µg/mL, respectively, compared with 41.68 ± 1.31 µg/mL in the control (*p* < 0.05). Consistent with this trend (Figure 2E,F), PC yield declined after day 4, reaching 97.45 ± 1.29 and 96.40 ± 1.44 mg/g at 3000 and 5000 mg/L, respectively, compared with 105.65 ± 1.47 mg/g in the control (*p* < 0.05). APC yield likewise declined to 22.44 ± 1.11 and 21.82 ± 1.14 mg/g, respectively, compared with 25.42 ± 1.22 mg/g in the control (*p* < 0.05).

### 3.3. Morphology and Surface Characteristics of A. platensis Under LDPE-MP Exposure

**Figure 3:**
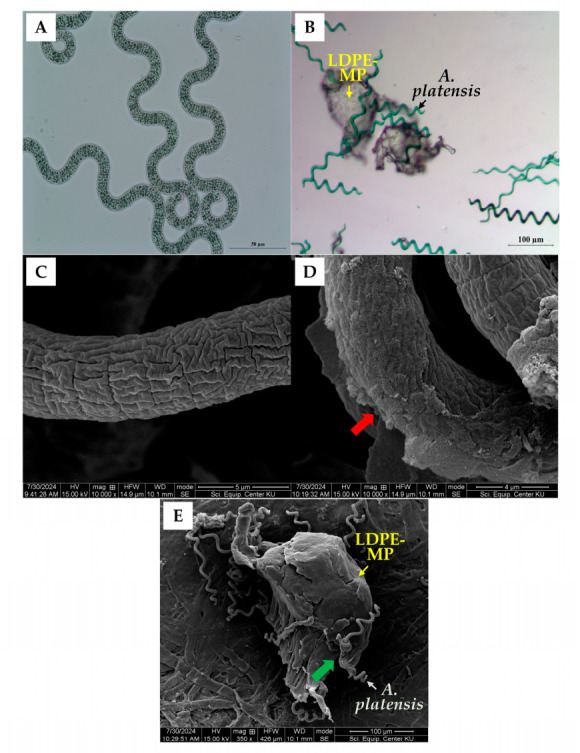
Microscopic observations of *A. platensis* after 6 days of LDPE-MP exposure (5000 mg/L; nominal size ≤ 500 µm). (**A**) Light micrograph of control cells without LDPE-MPs, and (**B**) cells exposed to LDPE-MPs. (**C**) FE-SEM image of a control filament, and (**D**) a filament following LDPE-MP exposure. (**E**) FE-SEM image illustrating the interaction between *A. platensis* and LDPE-MP fragments derived from a heterogeneous powder (nominal size ≤ 500 µm). A biofilm-like layer on the cell surface is indicated by the red arrow, while the green arrow marks smaller attached particles.

Light microscopy showed no apparent morphological differences in *A. platensis* between the control (0 mg/L) and the 5000 mg/L LDPE-MP treatment after 6 days (Figure 3A,B). In contrast, FE-SEM revealed a relatively smooth cell surface in the control (Figure 3C), whereas filaments in the treated group exhibited a thicker biofilm-like layer (Figure 3D, red arrow). In addition, smaller LDPE-MP fragments, derived from a heterogeneous powder (nominal size ≤ 500 µm), were observed attached to filament surfaces (Figure 3E, green arrow). These particles (approximately 100–200 µm) were observed in association with *A. platensis* filaments within the heterogeneous size distribution, consistent with localized heteroaggregate formation.

### 3.4. Identification of Differentially Expressed Genes

**Figure 4:**
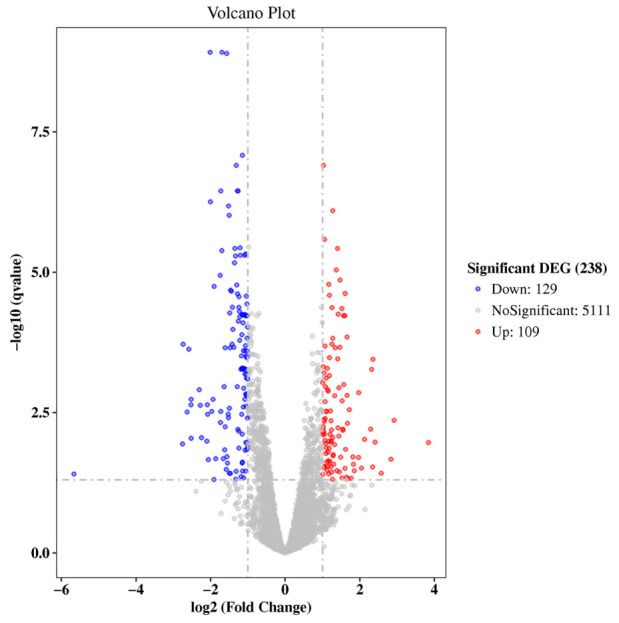
Volcano plot showing differential gene expression between the control group (0 mg/L) and the 5000 mg/L treatment group on day 6. Red dots indicate significantly upregulated DEGs, and blue dots indicate significantly downregulated DEGs. The *x*-axis represents the log_2_(Fold Change) in gene expression, and the *y*-axis represents the statistical significance of the differential expression on a log_10_(q value).

Bioinformatic analysis of the RNA-sequencing data identified 238 significantly differentially expressed genes (DEGs) between the control (0 mg/L) and the 5000 mg/L treatment groups (Figure 4), comprising 109 upregulated and 129 downregulated genes. Transcriptomic analysis revealed pronounced differential gene expressions in *A. platensis* following LDPE microplastic exposure. The most strongly upregulated DEGs encoded a pentapeptide repeat-containing protein, a molybdopterin oxidoreductase family protein, and a NUDIX hydrolase. Additional significantly upregulated genes included those encoding a class I S-adenosylmethionine (SAM)-dependent methyltransferase, the oxidative DNA damage-repair enzyme 8-oxo-dGTP diphosphatase (MutT), a ParA family protein, a nitrate ABC transporter permease, ferredoxin-nitrite reductase, a branched-chain amino acid ABC transporter permease, and exopolysaccharide biosynthesis protein. In contrast, the most strongly downregulated DEGs comprised genes encoding magnesium protoporphyrin IX methyltransferase and metallothionein, as well as (2Fe-2S) ferredoxin domain-containing protein. Further downregulated genes included Glu/Leu/Phe/Val dehydrogenase, a type IV pilin-like G/H family protein, and several serine proteases. A complete list of the 21 differentially expressed genes and their expression profiles is provided in Table 1.

### 3.5. Gene Ontology, Kyoto Encyclopedia of Genes and Genomes Pathway, and Cluster of Orthologous Groups Enrichment Analyses

**Figure 5:**
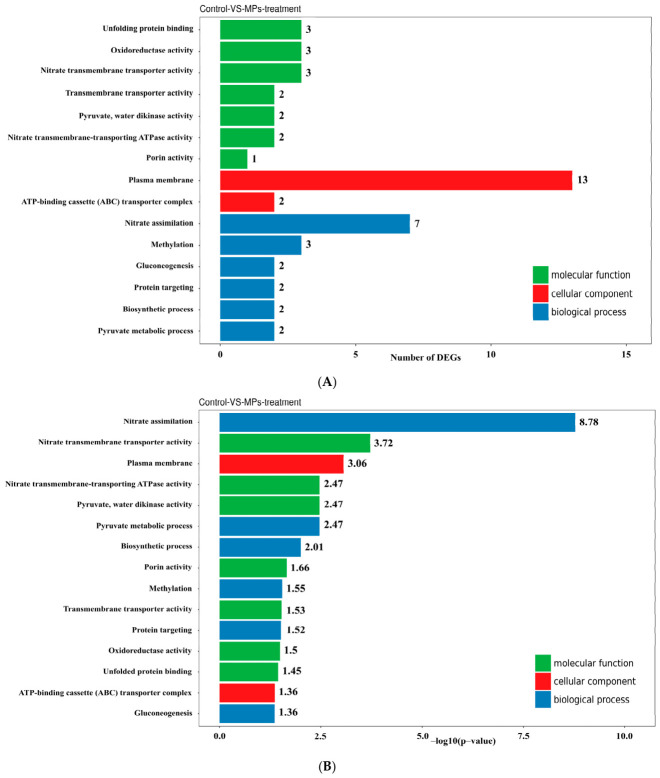
GO analysis of the DEGs. (**A**) Histogram of GO terms. The *x*-axis represents the number of DEGs associated with each GO category. Colors distinguish between the three GO domains: biological processes, cellular components, and molecular functions. (**B**) Histogram of GO enrichment *p*-values. The *x*-axis represents the −log_10_(*p*-value) for each term, and the *y*-axis displays the significantly enriched GO terms.

The DEGs were annotated with gene ontology (GO) terms and categorized into three main domains: biological process, cellular component, and molecular function (Figure 5A,B). The top 30 most enriched GO categories are presented. Within these domains, the annotated gene sequences were classified into 18 categories under biological process, 15 under cellular component, and 16 under molecular function. Notably, several enriched GO terms were associated with specific biological activities, including nitrate assimilation, nitrate transmembrane transporter activity, plasma membrane, nitrate transmembrane-transporting ATPase activity, pyruvate water dikinase activity, and pyruvate metabolic process.

**Figure 6:**
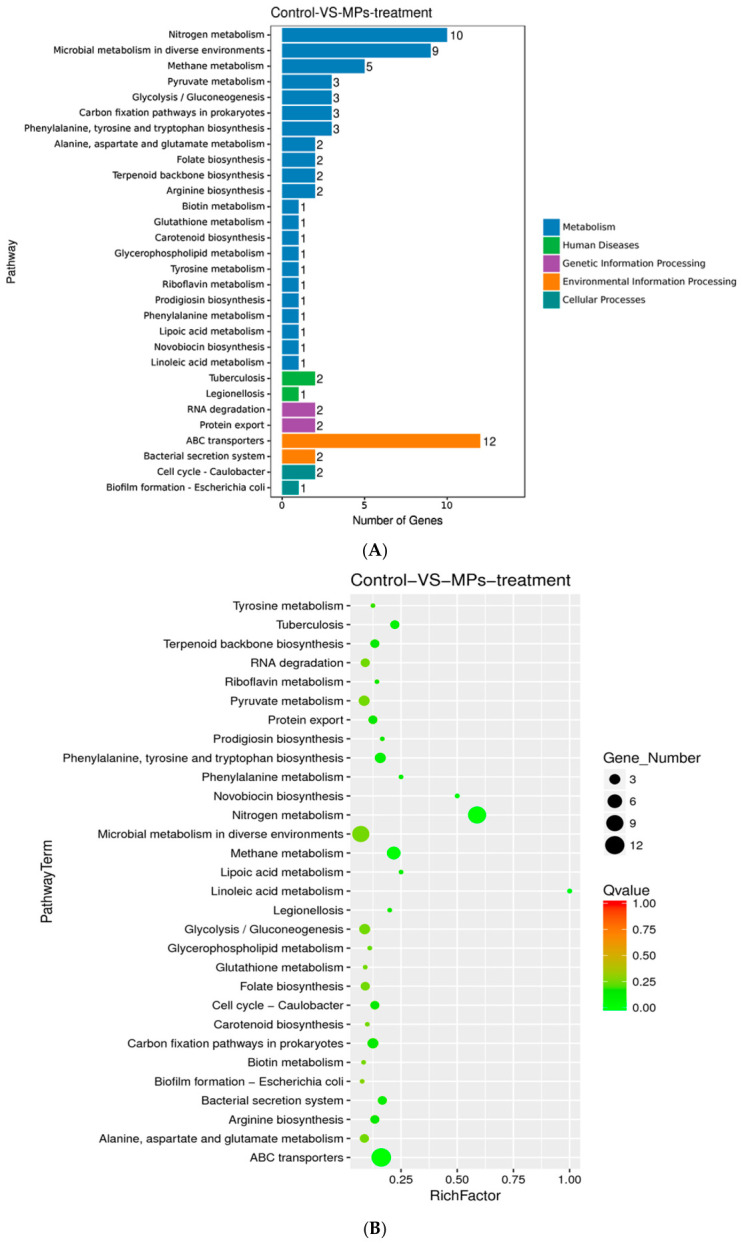
KEGG pathway analysis of DEGs. (**A**) Histogram of KEGG pathways. The *x*-axis represents the number of DEGs associated with each pathway, and the *y*-axis displays the KEGG pathway. Colors distinguish different pathway categories. (**B**) Scatter plot of KEGG pathway enrichment. The *x*-axis represents the Rich Factor, and the *y*-axis displays the KEGG Pathway Term. Dot size reflects the number of DEGs in each pathway, and color represents Q value ranges; the smaller the Q value, the more statistically significant the enrichment.

Kyoto Encyclopedia of Genes and Genomes (KEGG) pathway enrichment analysis revealed further insights. In the histogram (Figure 6A), the enriched pathways were categorized into five major functional groups: metabolism (55 DEGs), environmental information processing (14 DEGs), genetic information processing (4 DEGs), cellular processes (3 DEGs), and human diseases (3 DEGs). In the scatter plot (Figure 6B), KEGG enrichment was assessed based on three parameters: the rich factor, Q-value, and the number of DEGs mapped to each pathway. The rich factor is defined as the ratio of DEGs involved in each pathway to the total number of genes annotated in that pathway, where a higher rich factor indicates stronger enrichment. The Q value, a multiple testing-adjusted *p*-value, ranges from 0 to 1.00, with lower values indicating higher statistical significance. For further analysis, the top 30 enriched pathways were selected based on ascending Q values. If fewer than 30 pathways were identified, all were included in the analysis.

**Figure 7:**
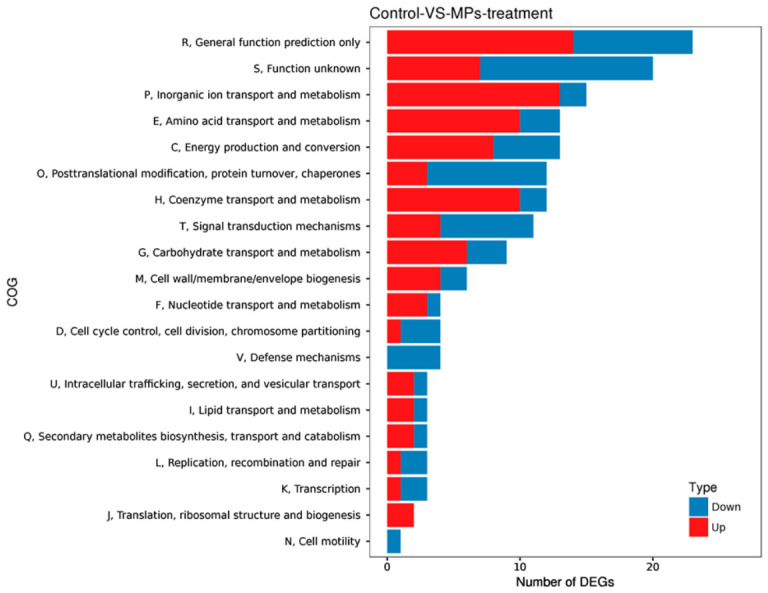
COG classification of DEGs. The histogram shows the distribution of DEGs across COG classes. The *x*-axis displays the number of DEGs, and the *y*-axis displays the COG classes. Red bars denote significantly upregulated DEGs, and blue bars denote significantly downregulated DEGs.

A Cluster of Orthologous Groups (COG) analysis was performed using the database curated by NCBI to classify protein functions based on evolutionary relationships among proteins encoded in bacterial, algal, and eukaryotic genomes. By aligning protein sequences with COGs, functional annotations were inferred from orthologous gene groupings. This analysis identified 93 genes as upregulated and 71 as downregulated (Figure 7). COG functional classification revealed that the most abundant categories among these DEGs included general function prediction only (25 DEGs), unknown function (23 DEGs), inorganic ion transport and metabolism (15 DEGs), energy production and conversion (13 DEGs), amino acid transport and metabolism (13 DEGs), coenzyme transport and metabolism (12 DEGs), and post-translational modification, protein turnover, and chaperones (12 DEGs).

## 4. Discussion

Although the LDPE-MP concentrations applied in this study exceed most reported environmental levels, they may represent potential worst-case scenarios, such as localized industrial discharge or accumulation in aquaculture systems. Considering the widespread use and environmental persistence of LDPE, fragmentation into microplastics may lead to localized hotspots with elevated concentrations in aquatic environments. Within this context, the present study systematically investigated how different concentrations of LDPE-MPs influence the physiological performance and gene expression of *A. platensis*, providing insight into concentration-dependent stress responses that may contribute to the observed growth inhibition. The current results showed that low to moderate concentrations of LDPE-MPs (10–1000 mg/L) with larger particle sizes (nominal size ≤ 500 µm) exerted minimal effects on the growth of *A. platensis*, consistent with a previous study reporting that polypropylene (PP) and high-density polyethylene (HDPE) microplastics (1000 mg/L) with particle sizes >400 µm did not exert an immediate effect on the growth of *Chlamydomonas reinhardtii* during the early stages of exposure [34]. In contrast, a previous study reported that LDPE-MPs of smaller size (<5 µm), even at low concentrations (1–100 mg/L), significantly inhibited the growth of *A. platensis* [20]. This discrepancy between particle sizes suggests that the biological effects of MPs may be size-dependent. Similarly, the growth rate of *Spirulina* sp. exposed to polyethylene terephthalate (PET) and polypropylene (PP) microplastics (1 µm in size) was lower than that of the control, with increasing microplastic concentrations (300–550 mg/L) associated with a reduction in growth rate of up to 75% [35]. Supporting this size-dependent effect, a previous study reported that polyethylene (PE) microplastics at a concentration of 250 mg/L inhibited the growth and reduced the biomass of *Scenedesmus quadricauda* more markedly when smaller particles (50 µm) were present compared with larger particles (nominal size ≤ 500 µm) after six days of exposure [36]. In addition, our results showed that growth inhibition occurred at high LDPE microplastic concentrations (3000–5000 mg/L) even with larger particles with a nominal size ≤ 500 µm, suggesting that microplastic concentration may play an important role under high exposure conditions. Overall, these findings indicate a potential size- and concentration-dependent response of *A. platensis* to microplastic exposure, where particle size appears to influence growth inhibition at lower concentrations, while at higher microplastic levels, concentration-related stress may become more pronounced. However, further studies using smaller-sized microplastics at high concentrations are needed to clarify whether concentration-dependent effects may outweigh those related to particle size.

Photosynthetic pigment composition in *A. platensis* showed concentration- and time-dependent changes during LDPE-MP exposure that were generally consistent with the observed trends in growth and biomass. At low to moderate concentrations (10–1000 mg/L), Chl *a*, PC, and APC remained statistically comparable to the control throughout the experiment, consistent with the minimal effects observed on growth and biomass. In contrast, higher LDPE-MP concentrations (3000–5000 mg/L) were associated with progressive declines in pigment levels. Reductions in Chl *a* contents became evident from day 4 onward, coinciding with the onset of growth inhibition and suggesting potential impacts on light harvesting and carbon assimilation [36,37]. Similarly, PC and APC levels decreased from day 4, which may reflect changes in the phycobilisome antenna system and reduced efficiency of energy transfer to the photosystems [19]. Additionally, the yields of Chl *a*, PC, and APC (normalized to biomass) were lower after day 4 at 3000 and 5000 mg/L MPs compared with the control, suggesting a possible reduction in pigment synthesis. Overall, these pigment responses indicate a concentration- and time-dependent effect of LDPE-MP exposure on the photosynthetic system of *A. platensis*, which may contribute to the observed reductions in growth and biomass.

While light microscopy revealed no visible morphological differences between control and 5000 mg/L LDPE-MP–treated *A. platensis* cells, FE-SEM analysis indicated noticeable surface-level changes at high microplastic concentrations. By day 6, treated filaments exhibited a thicker extracellular polymeric substance (EPS)-rich layer, with LDPE-MP particles occasionally attached to the surface. Smaller fragments (approximately 100–200 µm) were also observed within the heterogeneous size distribution, and were frequently associated with the filaments, consistent with heteroaggregate formation. These surface features were observed alongside the growth inhibition, biomass decline, and pigment reductions detected at higher LDPE-MP concentrations. EPS accumulation and the formation of microplastic–cell heteroaggregates may act as a physical barrier that potentially reduces light penetration and limits nutrient exchange between cells and the surrounding medium [34,38]. Such conditions may affect photosynthetic performance, consistent with the observed reductions in Chl *a*, PC, and APC from day 4. EPS-mediated aggregation may also contribute to localized shading and changes in the microenvironment around the cell surface, which could potentially affect the photosynthetic apparatus [39,40]. In addition, excessive EPS accumulation may restrict the diffusion of nutrients, inorganic carbon, and oxygen across the cell surface [41]. Previous studies have reported that *Spirulina* sp. can produce abundant EPS that readily interact with MPs to form heteroaggregates [42]. Moreover, previous studies have reported that 5 μm particles induced higher secretion of protein-rich EPS than smaller particles (0.065 and 0.5 μm) [43]. In the present study, the larger particle size (nominal size ≤ 500 µm) may provide additional surfaces for cell attachment, which could partly contribute to the EPS accumulation observed. Such physical interactions may be associated with the growth inhibition, biomass reduction, and pigment declines observed at higher LDPE-MP concentrations.

Transcriptomic analyses revealed that LDPE microplastic exposure disrupts multiple interconnected molecular pathways in *A. platensis*, providing mechanistic explanations for the observed concentration- and time-dependent reductions in growth, biomass, and photosynthetic pigments. The global transcriptional reprogramming indicates coordinated disturbances in nitrogen metabolism, central carbon metabolism, metal homeostasis, and photosynthetic redox balance. Enrichment of GO terms associated with nitrate assimilation, nitrate transmembrane transporter activity, and ATP-dependent ABC transporters suggests that nitrogen uptake and transmembrane nutrient transport were affected under LDPE-MP exposure. Although transcriptional upregulation of the nitrate ABC transporter permease was observed at high LDPE-MP concentrations, cellular growth remained markedly suppressed. This apparent inconsistency may reflect a compensatory transcriptional response to nitrogen limitation. However, EPS accumulation and microplastic–cell surface interactions may have reduced transporter efficiency and limited substrate diffusion, potentially contributing to the insufficient restoration of nitrate assimilation [44]. Further studies incorporating nutrient uptake kinetics are needed to confirm this mechanism.

In addition, the upregulation of exopolysaccharide biosynthesis-related proteins suggests enhanced EPS production in the treated cultures. In cyanobacteria such as *A. platensis*, EPS synthesis is commonly associated with stress responses and protective adaptations to unfavorable environmental conditions [33,34]. EPS can form a protective extracellular matrix surrounding cells, potentially reducing direct interactions with external stressors such as microplastic particles. Moreover, EPS may facilitate particle aggregation and surface attachment, which could further influence cell–particle interactions. Therefore, despite the observed reduction in cellular growth, the increased expression of EPS-related proteins may indicate a physiological shift from growth toward stress adaptation and cellular protection. Consistently, upregulation of molybdopterin oxidoreductase family members suggests an adaptive attempt to sustain nitrate reduction under LDPE-MP stress, as nitrate reductases coordinate nitrate reduction with nitrate/nitrite transport and photosynthetic electron flow in cyanobacteria, and increased expression of these enzymes may represent a compensatory response to stress-induced impairment of nutrient uptake and energy metabolism [45]. Nevertheless, concurrent downregulation of (2Fe–2S) ferredoxin domain-containing proteins indicates disruption of photosynthetic electron transfer, limiting reductant availability for nitrogen assimilation and carbon fixation [46].

Pigment depletion may be related to the downregulation of magnesium protoporphyrin IX methyltransferase, a key enzyme involved in chlorophyll biosynthesis [47]. Such transcriptional changes could contribute to the reductions in Chl *a* observed from day 4 at high LDPE-MP concentrations (3000–5000 mg/L). The subsequent declines in phycocyanin and allophycocyanin suggest that LDPE-MP exposure may influence multiple components of the photosynthetic system. At the genomic level, the upregulation of pentapeptide repeat-containing proteins and NUDIX hydrolases suggests a potential DNA protective response under LDPE-MP stress [48]. Additionally, enrichment of the pyruvate metabolic process may indicate a redistribution of carbon flux away from growth-related biosynthesis toward stress adaptation. KEGG pathway enrichment analysis further showed that many differentially expressed genes were associated with metabolic pathways, suggesting broader metabolic adjustments under LDPE-MP exposure. Consistently, the enrichment of COG categories related to energy production and conversion may reflect alterations in cellular energy metabolism, which could influence the energy available for cellular proliferation.

Metal homeostasis may also be affected, as indicated by the downregulation of metallothionein, a cysteine-rich protein involved in metal binding and detoxification [49]. Because trace metals function as essential cofactors in photosynthesis and nitrogen metabolism, alterations in their regulation may influence these processes. In parallel, downregulation of Glu/Leu/Phe/Val dehydrogenase may reflect changes in amino acid metabolic pathways, potentially affecting carbon and nitrogen incorporation into growth-related biosynthesis in cyanobacteria under microplastic exposure [50]. Reduced amino acid turnover may also influence protein synthesis and cellular energy metabolism. Under such conditions, carbon and nitrogen resources may be increasingly redirected toward EPS synthesis. Enhanced EPS production has been reported as a stress-related response that can facilitate microplastic binding and heteroaggregate formation [34,42]. Although EPS accumulation may initially reduce direct microplastic–cell interactions, it may also increase diffusion barriers for nutrients and impose additional metabolic demands on the cells [51]. Taken together, these findings provide insights into the physiological and transcriptomic responses of *A. platensis* to LDPE microplastic exposure. However, further studies are required to better elucidate the underlying mechanisms. Future research should include direct measurements of photosynthetic efficiency (e.g., Fv/Fm), reactive oxygen species (ROS), nutrient uptake kinetics, and metal quantification, as well as morphological parameters such as filament length, to improve understanding of how microplastics may influence cyanobacterial physiology.

## 5. Conclusions

This study investigated the effects of exposure to relatively high concentrations of LDPE microplastics (3000–5000 mg/L; nominal size ≤ 500 µm) on the growth and physiological responses of *Arthrospira platensis*. Growth suppression and reduced biomass accumulation were observed in a concentration- and time-dependent manner, accompanied by declines in photosynthetic pigments and indications of potential changes in the photosynthetic system. Microscopic observations showed adhesion of LDPE particles to filament surfaces and the formation of EPS-rich biofilms, which may influence light availability and nutrient exchange around the cells. Transcriptomic analysis further revealed stress-associated responses, including the upregulation of defense-related genes and the downregulation of genes involved in electron transfer, metal homeostasis, and energy metabolism. Overall, these results suggest that exposure to high levels of LDPE microplastics may affect multiple physiological processes in *A. platensis*. However, additional studies under environmentally relevant conditions are required to further clarify the mechanisms and ecological implications of microplastic exposure for cyanobacteria.

## Figures and Tables

**Table 1:** Summary of differentially expressed genes (DEGs) in response to MP exposure.

Gene_ID	Log_2_Foldchange (Control/MP Treatment)	*p*-Value	Regulation	Product
gene-GDL46_RS21515	3.843323037	1.18 × 10^−3^	Ups	Pentapeptide repeat-containing protein
gene-GDL46_RS10045	2.921408004	3.26 × 10^−4^	Ups	Molybdopterin oxidoreductase family protein
gene-GDL46_RS09345	2.837194691	2.96 × 10^−3^	Ups	NUDIX hydrolase
gene-GDL46_RS12180	2.576470833	6.69 × 10^−3^	Ups	Class I SAM-dependent methyltransferase
gene-GDL46_RS08040	2.410200605	1.15 × 10^−3^	Ups	8-oxo-dGTP diphosphatase MutT
gene-GDL46_RS06185	2.344934354	4.71 × 10^−3^	Ups	ParA family protein
gene-GDL46_RS08610	2.319049407	1.80 × 10^−5^	Ups	Alpha/beta fold hydrolase
gene-GDL46_RS10040	2.127984167	9.67 × 10^−4^	Ups	MFS transporter
gene-GDL46_RS24255	2.047222623	5.03 × 10^−3^	Ups	Nitrate ABC transporter permease
gene-GDL46_RS12275	1.966955189	2.65 × 10^−3^	Ups	Ferredoxin—nitrite reductase
gene-GDL46_RS15090	1.852646941	5.84 × 10^−3^	Ups	Ferredoxin
gene-GDL46_RS13530	1.830867459	4.03 × 10^−3^	Ups	Branched-chain amino acid ABC transporter permease
gene-GDL46_RS19045	1.572226911	4.13 × 10^−5^	Ups	Exopolysaccharide biosynthesis protein
gene-GDL46_RS10385	−1.933254634	9.45 × 10^−5^	Down	(2Fe-2S) ferredoxin domain-containing protein
gene-GDL46_RS07465	−1.967483465	1.92 × 10^−4^	Down	COP23 domain-containing protein
gene-GDL46_RS05620	−2.011441718	6.64 × 10^−13^	Down	Type II toxin-antitoxin system HicA family toxin
gene-GDL46_RS04890	−2.059968354	3.02 × 10^−3^	Down	23S ribosomal RNA
gene-GDL46_RS07460	−2.079463251	2.29 × 10^−4^	Down	Serine protease
gene-GDL46_RS07455	−2.100216518	1.08 × 10^−3^	Down	Serine protease
gene-GDL46_RS25035	−2.244360024	8.79 × 10^−4^	Down	Tetratricopeptide repeat protein
gene-GDL46_RS16410	−2.276506487	1.32 × 10^−4^	Down	Glu/Leu/Phe/Val dehydrogenase
gene-GDL46_RS00660	−2.304786654	5.52 × 10^−5^	Down	Type IV pilin-like G/H family protein
gene-GDL46_RS17105	−2.519496853	9.42 × 10^−5^	Down	Magnesium protoporphyrin IX methyltransferase
gene-GDL46_RS15220	−2.524770615	1.28 × 10^−4^	Down	Metallothionein
gene-GDL46_RS17110	−2.735504189	4.16 × 10^−6^	Down	PspC domain-containing protein

## Data Availability

The original contributions presented in the study are included in the article, further inquiries can be directed to the corresponding authors.

## References

[1] Boctor J., Hoyle F.C., Farag M.A., Ebaid M., Walsh T., Whiteley A.S., Murphy D.V. (2025). Microplastics and nanoplastics: Fate, transport, and governance from agricultural soil to food webs and humans. Environ. Sci. Eur..

[2] Rai M., Pant G., Pant K., Aloo B.N., Kumar G., Singh H.B., Tripathi V. (2023). Microplastic pollution in terrestrial ecosystems and its interaction with other soil pollutants: A potential threat to soil ecosystem sustainability. Resources.

[3] Bashir I., Lone F.A., Bhat R.A., Mir S.A., Dar Z.A., Dar S.A. (2020). Concerns and threats of contamination on aquatic ecosystems. Bioremediat. Biotechnol..

[4] Menéndez-Pedriza A., Jaumot J. (2020). Interaction of environmental pollutants with microplastics: A critical review of sorption factors, bioaccumulation and ecotoxicological effects. Toxics.

[5] Klein S., Worch E., Knepper T.P. (2015). Occurrence and spatial distribution of microplastics in river shore sediments of the Rhine-Main area in Germany. Environ. Sci. Technol..

[6] Alomar C., Estarrellas F., Deudero S. (2016). Microplastics in the Mediterranean Sea: Deposition in coastal shallow sediments, spatial variation and preferential grain size. Mar. Environ. Res..

[7] Kumar Sen S., Raut S. (2015). Microbial degradation of low density polyethylene (LDPE): A review. J. Environ. Chem. Eng..

[8] Andrady A.L., Neal M.A. (2009). Applications and societal benefits of plastics. Philos. Trans. R. Soc. Lond. B Biol. Sci..

[9] Gowthami A., Syed Marjuk M., Raju P., Nanthini Devi K., Santhanam P., Dinesh Kumar S., Perumal P. (2023). Biodegradation efficacy of selected marine microalgae against low-density polyethylene (LDPE): An environment friendly green approach. Mar. Pollut. Bull..

[10] Li J., Liu H., Paul Chen J. (2018). Microplastics in freshwater systems: A review on occurrence, environmental effects, and methods for microplastics detection. Water Res..

[11] McCormick A.R., Hoellein T.J., London M.G., Hittie J., Scott J.W., Kelly J.J. (2016). Microplastic in surface waters of urban rivers: Concentration, sources, and associated bacterial assemblages. Ecosphere.

[12] Biswas B., Joseph A., Goel S. (2016). Microplastics in river water: Occurrence, weathering, and adsorption behaviour. Environ. Sci. Water Res. Technol..

[13] Song Y.K., Hong S.H., Jang M., Kang J.H., Kwon O.Y., Han G.M., Shim W.J. (2014). Large accumulation of micro-sized synthetic polymer particles in the sea surface microlayer. Environ. Sci. Technol..

[14] Lasee S., Mauricio J., Thompson W.A., Karnjanapiboonwong A., Kasumba J., Subbiah S., Morse A.N., Anderson T.A. (2017). Microplastics in a freshwater environment receiving treated wastewater effluent. Integr. Environ. Assess. Manag..

[15] Eydi Gabrabad M., Yari M., Bonyadi Z. (2024). Using *Spirulina platensis* as a natural biocoagulant for polystyrene removal from aqueous medium: Performance, optimization, and modeling. Sci. Rep..

[16] Yang Y., Zhang X., Tan L., Xin R., Ma Y., Niu Z. (2026). Interactions between cyanobacteria and emerging contaminants in aqueous environments. Aquat. Toxicol..

[17] Chang M., Liu K. (2023). *Arthrospira platensis* as future food: A review on functional ingredients, bioactivities and application in the food industry. Int. J. Food. Sci. Technol..

[18] Gentscheva G., Nikolova K., Panayotova V., Peycheva K., Makedonski L., Slavov P., Radusheva P., Petrova P., Yotkovska I. (2023). Application of *Arthrospira platensis* for medicinal purposes and the food industry: A review of the literature. Life.

[19] Hadiyanto H., Khoironi A., Dianratri I., Suherman S., Muhammad F., Vaidyanathan S. (2021). Interactions between polyethylene and polypropylene microplastics and *Spirulina* sp. microalgae in aquatic systems. Heliyon.

[20] Abbasi S., Amiranipour S., Karimi J., Mohsenzadeh S., Turner A. (2023). Impacts of polyethylene microplastics on the microalga, Spirulina (*Arthrospira platensis*). Environ. Pollut..

[21] De Sá Silva C.A., De Andrade N.J., de Fatima Ferreira Soares N., Ferreira S.O. (2003). Evaluation of ultraviolet radiation to control microorganisms adhering to low-density polyethylene films. Braz. J. Microbiol..

[22] Ogodo A.C., Agwaranze D.I., Daji M., Aso R.E. (2022). Microbial techniques and methods: Basic techniques and microscopy. Analytical Techniques in Biosciences: From Basics to Applications.

[23] Promariya A., Treenarat S., Akrimajirachoote N., Sricharern W., Raksajit W. (2025). Cultivation of *Arthrospira platensis* in veterinary hospital wastewater enhances pigment production and reduces antibiotic resistance genes. Biology.

[24] Kim G.-H., Lee Y.J., Kwon J.-H. (2025). Relationship between harvesting efficiency and filament morphology in *Arthrospira platensis* Gomont. Microorganisms.

[25] Ritchie R.J. (2006). Consistent sets of spectrophotometric chlorophyll equations for acetone, methanol and ethanol solvents. Photosynth. Res..

[26] Hotos G.N., Antoniadis T.I. (2022). The Effect of colored and white light on growth and phycobiliproteins, chlorophyll and carotenoids content of the marine cyanobacteria *Phormidium* sp. and *Cyanothece* sp. in batch cultures. Life.

[27] Khandual S., Sanchez E., Andrews H., de la Rosa J. (2021). Phycocyanin content and nutritional profile of *Arthrospira platensis* from Mexico: Efficient extraction process and stability evaluation of phycocyanin. BMC Chem..

[28] Tavanandi H.A., Chandralekha Devi A., Raghavarao K. (2018). A newer approach for the primary extraction of allophycocyanin with high purity and yield from dry biomass of *Arthrospira platensis*. Sep. Purif. Technol..

[29] Pathak R.R., Lochab S. (2010). A method for rapid isolation of total RNA of high purity and yield from *Arthrospira platensis*. Can. J. Microbiol..

[30] Bolger A.M., Lohse M., Usadel B. (2014). Trimmomatic: A flexible trimmer for Illumina sequence data. Bioinformatics.

[31] Love M.I., Huber W., Anders S. (2014). Moderated estimation of fold change and dispersion for RNA-seq data with DESeq2. Genome Biol..

[32] Young M.D., Wakefield M.J., Smyth G.K., Oshlack A. (2010). Gene ontology analysis for RNA-seq: Accounting for selection bias. Genome Biol..

[33] Yu G., He Q.Y. (2016). ReactomePA: An R/Bioconductor package for reactome pathway analysis and visualization. Mol. Biosyst..

[34] Lagarde F., Olivier O., Zanella M., Daniel P., Hiard S., Caruso A. (2016). Microplastic interactions with freshwater microalgae: Hetero-aggregation and changes in plastic density appear strongly dependent on polymer type. Environ. Pollut..

[35] Khoironi A., Anggoro S., Sudarno S. (2019). Evaluation of the interaction among microalgae *Spirulina* sp., plastics polyethylene terephthalate and polypropylene in freshwater environment. J. Ecol. Eng..

[36] Wang Y., Xie F., Li W., Ji L., Guan G., Abudula A., Yang Z., Gao F. (2025). Impact of microplastics on growth and lipid accumulation in *Scenedesmus quadricauda*. Fermentation.

[37] Zhao M., Ren Z., Wei Z., Shi H., Wang L., Liang Y. (2024). The effect of polyethylene microplastics on growth and antioxydant response of *Oscillatoria princeps* and *Chlorella pyrenoidosa*. Bull. Environ. Contam. Toxicol..

[38] Zhang C., Chen X., Wang J., Tan L. (2017). Toxic effects of microplastic on marine microalgae *Skeletonema costatum*: Interactions between microplastic and algae. Environ. Pollut..

[39] Song Y., Zhang B., Si M., Chen Z., Geng J., Liang F., Xi M., Liu X., Wang R. (2023). Roles of extracellular polymeric substances on *Microcystis aeruginosa* exposed to different sizes of polystyrene microplastics. Chemosphere.

[40] Yue Z., Qian J., Li W., Liu X., Dai H., Liu X., Pi F., Wang J. (2025). Spotlight on the long-term effects of micro/nanoplastics exposure on *Spirulina platensis*: Algal cells, extracellular polymeric substances, and phycocyanin. Food Chem..

[41] Zhou Y., Cui X., Wu B., Wang Z., Liu Y., Ren T., Xia S., Rittmann B.E. (2024). Microalgal extracellular polymeric substances (EPS) and their roles in cultivation, biomass harvesting, and bioproducts extraction. Bioresour. Technol..

[42] Hadiyanto H., Khoironi A., Dianratri I., Joelyna F.A., Christwardana M., Sabhira A.I., Baihaqi R.A. (2025). Microplastic removal in aquatic systems using extracellular polymeric substances (EPS) of microalgae. Sustain. Environ..

[43] Gong X., Ge Z., Ma Z., Li Y., Huang D., Zhang J. (2023). Effect of different size microplastic particles on the construction of algal-bacterial biofilms and microbial communities. J. Environ. Manag..

[44] Fang X.Z., Fang S.Q., Ding Y., Ma J.W., Ye Z.Q., Liu D., Zhao K.L. (2024). Microplastic exposure inhibits nitrate uptake and assimilation in wheat plants. Environ. Pollut..

[45] Gan Y., Gong B., Huang X., Fang F., Peng T., Liu Z. (2024). Response of aerobic granular sludge under acute inhibition by polystyrene microplastics: Activity, aggregation performance, and microbial analysis. Environ. Pollut..

[46] Cassier-Chauvat C., Chauvat F. (2014). Function and regulation of ferredoxins in the cyanobacterium, *Synechocystis* PCC6803: Recent advances. Life.

[47] Zheng X., Liu X., Zhang L., Wang Z., Yuan Y., Li Y., Li Y., Huang H., Cao X., Fan Z. (2022). Toxicity mechanism of Nylon microplastics on *Microcystis aeruginosa* through three pathways: Photosynthesis, oxidative stress and energy metabolism. J. Hazard. Mater..

[48] Okuda K., Nishiyama Y., Morita E.H., Hayashi H. (2004). Identification and characterization of NuhA, a novel Nudix hydrolase specific for ADP-ribose in the cyanobacterium *Synechococcus* sp. PCC 7002. Biochim. Biophys. Acta.

[49] Mikhaylina A., Scott L., Scanlan D.J., Blindauer C.A. (2022). A metallothionein from an open ocean cyanobacterium removes zinc from the sensor protein controlling its transcription. J. Inorg. Biochem..

[50] Xiao X., Li W., Li S., Zuo X., Liu J., Guo L., Lu X., Zhang L. (2023). The growth inhibition of polyethylene nanoplastics on the bait-microalgae *Isochrysis galbana* based on the transcriptome analysis. Microorganisms.

[51] Rossi F., De Philippis R. (2015). Role of cyanobacterial exopolysaccharides in phototrophic biofilms and in complex microbial mats. Life.

